# Computed Tomography in the Evaluation and Identification of Features of Coronary Atherosclerosis Between European and Asian Populations in Kazakhstan

**DOI:** 10.3390/medicina62030527

**Published:** 2026-03-12

**Authors:** Tairkhan Dautov, Elmira Yelshibayeva, Makhabbat Tynybekova, Bakyt Duisenbayeva, Lazzat Bastarbekova, Tokhirzhan Tashpulatov, Kuralay Sharipova, Shokhrukh Akhnazarov, Daniyar Kudabayev, Kemelya Nigmetova, Nurly Kapashova

**Affiliations:** 1 Department of Diagnostic and Interventional Radiology, Heart Center, Astana 010000, Kazakhstan; tairkhan.dautov@gmail.com (T.D.); 1bastarbekova@mail.ru (L.B.); 2 RSE “Medical Center Hospital of the President’s Affairs Administration of the Republic of Kazakhstan”, Astana 010000, Kazakhstan; makhabbattynybekova@gmail.com (M.T.); tashpulatov_t@inbox.ru (T.T.); shax.akhnazarov@gmail.com (S.A.); kudabayev.dk@gmai.com (D.K.); kemelya12345@gmail.com (K.N.); 3 Department of Diagnostic Radiology, University Medical Center, Astana 010000, Kazakhstan; bakytduisenbayeva@gmail.com; 4 Department of Human Anatomy Named After A.B. Aubakirov, NpJSC “Astana Medical University”, Astana 010000, Kazakhstan; kaya1974@mail.ru; 5 School of Medicine, Nazarbayev University, Astana 010000, Kazakhstan; nurly.kapashova@nu.edu.kz

**Keywords:** coronary CT angiography, coronary artery disease, calcium score, European and Asian population, ethnic differences, coronary atherosclerosis

## Abstract

*Background and Objectives*: This study aimed to compare coronary plaque characteristics between Asian and European populations undergoing coronary CT angiography and to examine associations between cardiovascular risk factors and coronary artery calcification. *Materials and Methods*: In this retrospective, two-center, cross-sectional observational study, 1591 adult patients (1203 of Asian and 388 of European descent) referred for coronary computed tomography angiography (CCTA) due to suspected coronary artery disease between 2008 and 2025 were included. Demographic, clinical characteristics, and laboratory data were obtained from medical records. Computed tomography (CT) was performed on different CT scanners, including a 64-slice Siemens SOMATOM Definition AS, a 250-slice Siemens SOMATOM, a 640-slice multi-detector Canon Aquilion ONE, and a 128-slice multi-detector GE Revolution scanner with prospective cardiac synchronization and 0.6 mm slice reconstruction. Coronary artery calcium (CAC) scores were quantified using automated software “Vitrea”. Associations between ethnicity, cardiovascular risk factors, and CAC were assessed using non-parametric analyses and multivariable regression models. Stata 18 software was used for all statistical analyses. *Results*: European participants demonstrated a higher prevalence of obesity, hypertension, tobacco use, and alcohol consumption compared with Asian participants. The prevalence of CAC > 0 was higher in Europeans than in Asians (60.6% vs. 50.3%, *p* < 0.01). European individuals were independently associated with CAC presence in multivariable analysis. Multivessel (≥2-vessel) stenosis and calcified plaques were more frequently observed in Europeans, whereas non-calcified and low-density plaques predominated among Asians. *Conclusions*: Within this referral-based cohort, differences in coronary plaque characteristics were observed between the studied groups within this clinical CCTA cohort. The European group was associated with a higher prevalence of calcified plaques, whereas non-calcified and low-density plaques were more frequently observed among Asian participants. These findings show associations between ethnicity and plaque characteristics within a clinical cohort and require confirmation in prospective studies.

## 1. Introduction

Cardiovascular diseases (CVD), which include ischemic heart disease (IHD), stroke, heart failure, peripheral arterial disease, arrhythmia, and valvular disease, remain the leading cause of mortality worldwide [1]. According to the European Society of Cardiology, approximately one in three deaths in the European Union is attributed to CVD, corresponding to nearly 4600 deaths per day. In 2025, an estimated 62 million individuals in Europe are living with CVD [2]. The most recent Global Burden of Disease (GBD) analysis reported 19.2 million CVD deaths in 2023, accounting for 46.6% of global CVD mortality [3].

Although age-standardized CVD mortality rates among young adults have remained stable, hypertension-related CVD mortality has increased by 78.5%, suggesting a shift in the onset of CVD toward earlier ages and an increasing number of coronary events among young adults with hypertension [4]. This trend is primarily driven by the increased frequency of modifiable risk factors at a young age, such as hypertension, obesity, diabetes, tobacco use, alcohol consumption, and poor dietary habits that promote atherogenic lipid profiles and accelerate atherosclerosis [5]. Over the past three decades, the adult population aged 30–79 years living with hypertension has risen from 650 million to 1.28 billion, while about 580 million individuals (41% of women and 51% of men) remain unaware of their condition due to a lack of diagnosis [6].

Obesity represents one of the major contributors to cardiovascular mortality, accounting for more than 13% of deaths in the European region. In 2019, 17% of people in European Society of Cardiology (ESC) member countries were classified as obese, and 55% were overweight. Regular tobacco use increases the risk of fatal CVD events by up to threefold, and in some ESC member countries, smoking prevalence is close to 50%, especially in parts of Eastern Europe and Central Asia [7]. Alcohol consumption rates have also increased, and excessive intake has been associated with cardiovascular disease, alcohol-associated cirrhosis, and liver cancer among adults aged 15–49 years in 2024 [8].

Over the past decade, calcified vascular lesions have been widely used as a reliable indicator of CVD severity and have been shown to correlate with future coronary events in large studies such as the Multi-Ethnic Study of Atherosclerosis (MESA) [9,10].

However, the distribution and predictive significance of CAC may vary by race. For example, research has shown significant variation in CAC burden and plaque composition among distinct ethnic groups [11,12]. Understanding such variability is important, as exclusive reliance on CAC scoring may underestimate cardiovascular risk in populations with a higher prevalence of non-calcified plaque.

Importantly, the early emergence of CVD risk factors in young adults is often underestimated. Reliance solely on CAC scoring may fail to identify individuals with high-risk non-calcified plaques, potentially delaying risk stratification and preventive interventions. As a result, premature CVD events and mortality rates continue to increase in this population. Therefore, the purpose of this study was to compare these differences using coronary CT angiography (CCTA) and to evaluate differences in plaque characteristics beyond CAC scoring within this clinical cohort.

According to the 2024 ESC guidelines for chronic coronary syndromes, CCTA is recommended as a primary non-invasive anatomical imaging modality for patients with suspected CAD and low or moderate pre-test probability of obstructive CAD (5–50%) to diagnose obstructive lesions and estimate the risk of major adverse cardiovascular events (MACE) [13]. In comparison, reports from the Asian Society of Cardiovascular Imaging-Practical Tutorial (ASCI-PT) focus on standardized evaluation of stenosis and plaque features using CCTA to guide clinical decision-making strategies [14]. Assessing potential differences in plaque morphology within demographically diverse populations, such as those in Central Asia, may contribute to improved characterization of coronary atherosclerosis.

## 2. Materials and Methods

### 2.1. Study Design and Setting

This retrospective, two-center, cross-sectional observational study was conducted at the Medical Centre Hospital of the President’s Affairs Administration and the Heart Center University Medical Center (Heart Center UMC), both of which provide healthcare services to patients across Kazakhstan. All patients were registered in a dedicated, autonomous hospital database maintained exclusively by these centers. These centers primarily provide healthcare services to individuals employed in the public sector, representing a population with average socioeconomic status. Informed consent was obtained in three languages (Kazakh, Russian, and English; depending on the racial and ethnic background of patients) from all patients before CT examination, permitting the use of their collected medical data for research purposes. A total of 1591 consecutive adult patients referred for coronary CT angiography (CCTA) for evaluation of suspected coronary artery disease were included. Medical records for the period of 2008 to 2025 were retrospectively reviewed.

### 2.2. Study Population

Adult patients aged >18 years who were referred for coronary CT angiography (CCTA) for evaluation of suspected coronary artery disease were eligible for inclusion. Four age groups were used to stratify the patients: <50, 51–60, 61–70, and >70 years. Demographic characteristics, including age, gender, body mass index (BMI) as well as coronary heart disease (CHD) risk factors included: the highest recorded systolic (SBP) and diastolic blood pressure (DBP) values; previously diagnosed hypertension or systolic blood pressure exceeding 140 mm of Hg or diastolic blood pressure exceeding 90 mm of Hg; tobacco use (yes or no); alcohol consumption (any amount of alcohol drinks, yes or no); family history of CVD (first or second-degree relative with CVD); diabetes mellitus (I or II type) were obtained from medical records. Laboratory parameters, including plasma glucose ≥ 7 mmol/L, plasma fibrinogen level, serum creatinine, a full lipid profile (total cholesterol, low-density lipoprotein cholesterol (LDL-C), and high-density lipoprotein cholesterol (HDL-C)), and triglyceride concentration), were recorded. The chest pain was grouped as typical, atypical, and non-anginal.

Among the 1591 patients, 1203 were of Asian descent (Kazakh, Tatar, Uzbek, Uyghyr, Kyrgyz, Tajik, Turkmen, Bashkir) and 388 were of European descent (Ukrainian, German, Belarus, Russian, Polish, Slovak).

### 2.3. Clinical Management and Lifestyle Measures

Medication history was reviewed. Patients with established CVD or hypertension were prescribed statin therapy according to ESC guidelines, as lifelong and dose-titrated individually based on lipid levels [15]. The exact cumulative dose in each patient was not analyzed; only the treatment scheme was documented. Antihypertensive treatment was prescribed according to ESC guidelines and included such pharmacological classes as Angiotensin-Converting Enzyme inhibitors (ACEi), Angiotensin II Receptor Blockers (ARBs), dihydropyridine calcium channel blockers (CCBs), diuretics, potassium-sparing diuretics, and beta-blockers [16]. Due to the retrospective design and long period of the study, a complete quantitative distribution of individual drug classes on 1591 patients was not consistently available. Dietary recommendations depending on comorbidities and based on the Pevzner therapeutic diet classification, a system historically used in regional clinical practice were prescribed. These dietary prescriptions (diet No 10: low-salt, low-fat diet for cardiovascular disease and diet No 9: carbohydrate-controlled diet for diabetes) are similar to contemporary ESC guidelines for cardiovascular and diabetes management. All patients were prescribed regular physical activity based on the ESC guidelines for cardiovascular risk reduction. The implementation of prescribed physical activity was monitored at three-month intervals during standard cardiology check-ups [15].

### 2.4. Clinical Characteristics and Anatomical Risk Stratification

Risk stratification was performed according to a modified risk assessment based on the ESC guidelines for coronary artery disease [16]. Due to the retrospective nature of the study and available data limitations, risk stratification was based on specific laboratory, clinical results, and anatomical assessment consisting of coronary artery stenosis severity. Clinical risk assessment included the presence of arterial hypertension and characterization of chest pain as typical angina, atypical angina, and non-anginal chest pain. Anatomical risk stratification was based on CT-angiography findings. Obstructive coronary artery disease (CAD) was defined as ≥50% luminal stenosis, while <50% was considered non-obstructive. Other ESC parameters, such as exercise testing, echocardiographic data, and functional imaging, were not included in this study.

### 2.5. Imaging Systems

Computed tomography (CT) was performed using a 64-slice Siemens SOMATOM Definition AS (Siemens Healthineers, Erlangen, Germany) scanner between 2008 and 2014, and a 250-slice Siemens SOMATOM Force (Siemens Healthineers, Erlangen, Germany) scanner from 2015 to 2022. From 2022 to the present, CT imaging was conducted using a 640-slice multi-detector Canon Aquilion ONE (Canon Medical Systems, Otawara, Japan), a 250-slice Siemens SOMATOM Force (Siemens Healthineers, Erlangen, Germany), and a 128-slice multi-detector GE Revolution (GE Healthcare, Chicago, IL, USA) scanner with prospective cardiac synchronization and 0.6 mm slice reconstruction.

The Agatston calcium score was calculated using automated software (Calcium Scoring, Vitrea, version 7.14 (Vital Images, Minneapolis, MN, USA)) with calcified lesions defined as areas with a minimal area of ≥1 and a density ≥ 130 Hounsfield Units (HU).

Coronary computed tomographic angiography (CCTA) imaging was performed following intravenous administration of iodinated contrast medium (Visipaque 320 mL (GE Healthcare, Chicago, IL, USA) and Ultravist 370 mL (Bayer Healthcare, Leverkusen, Germany)). A volume of 100 mL was used on average, and the actual dose was calculated based on each patient’s body weight and injected at an infusion rate of 4 and 4.5–5.0 mL/s, depending on the contrast agent.

Contrast-enhanced coronary CT-angiography was performed using three different CT scanners. Based on ESC protocols for coronary CT-angiography, the effective radiation dose was estimated to range from approximately 8 to 13 mSv per examination in the period between 2022 and the present and 17 to 25 mSv per examination in the period from 2008 to 2022, depending on scanner type and acquisition protocol [16].

### 2.6. Ethics

This study was conducted in accordance with ethical approval obtained from the Hospital’s Local Bioethics Committee (Institutional Review Boards), as stated in Protocol No.7 dated 9 December 2025, and in accordance with the Declaration of Helsinki.

### 2.7. Statistical Analysis

The distribution of continuous variables was assessed using histograms and summary statistics, including skewness and kurtosis. Variables demonstrating approximately symmetric distributions were summarized as mean ± standard deviation (SD), whereas variables with right-skewed distributions were summarized as median with interquartile range (IQR).

To evaluate differences in demographic characteristics and CHD risk factors between racial groups, Student’s *t*-test was used for normally distributed continuous variables and the Mann–Whitney U test for non-normally distributed continuous variables. For comparisons involving more than two groups the Kruskal–Wallis test was applied. Categorical variables were compared using Pearson’s chi-square test or Fisher’s exact test. Data were screened for implausible values and extreme outliers before analysis.

The coronary artery calcium (CAC) score demonstrated a markedly right-skewed distribution with a substantial proportion of zero values. Accordingly, a two-part modeling strategy was applied. First, non-parametric tests (Mann–Whitney U test, Kruskal–Wallis test, and Spearman’s correlation coefficient) were used to evaluate bivariate associations involving the continuous CAC score. Second, the CAC score was dichotomized (CAC > 0 vs. CAC = 0), and multivariable logistic regression analysis was performed to assess factors associated with CAC presence, adjusting for potential confounders [12]. Because the assumption of linearity in the logit was violated, systolic blood pressure and total cholesterol were categorized in the logistic regression model. Among participants with CAC > 0, multivariable linear regression analysis was conducted using natural log-transformed CAC values to evaluate determinants of CAC burden. Clinically relevant cardiovascular risk factors were included in multivariable models irrespective of univariate statistical significance to minimize residual confounding.

The multivariable logistic regression model for CAC presence included ethnicity, age category, sex, BMI, smoking status, alcohol use, diabetes, systolic blood pressure category, total cholesterol category, statin use, and family history of coronary heart disease.

Analyses were conducted using complete case analysis. All statistical tests were two-sided, and a *p*-value < 0.05 was considered statistically significant. Statistical analyses were performed using Stata version 18.

## 3. Results

### 3.1. Demographics, Behavioral Habits, and Laboratory Profiles by Race

**Figure 1:**
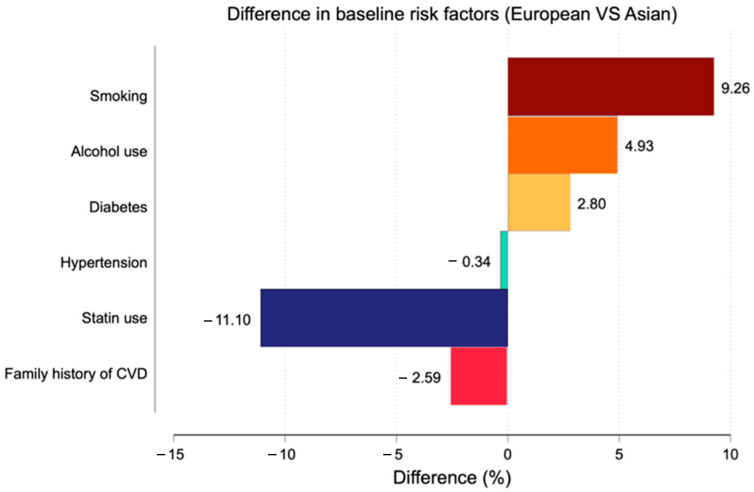
Baseline cardiovascular risk factors in European and Asian participants. Variables included smoking, alcohol use, diabetes, hypertension, statin use, and family history of cardiovascular disease (CVD).

The study population included 1591 patients, comprising 1203 Asian and 388 European individuals. European participants were slightly older and had a significantly higher proportion of individuals aged over 60 years compared with Asians (*p* < 0.01) (Table 1). Several cardiovascular risk factors differed significantly between race groups (Figure 1).

European participants had higher BMI (*p* < 0.01), higher systolic (*p* = 0.01) and diastolic blood pressure (*p* < 0.01), and a higher prevalence of smoking (*p* < 0.01) and alcohol consumption (*p* < 0.01), whereas statin use was more frequent among Asian participants (49.5%, *p* < 0.01). No significant differences were observed in laboratory parameters, except for higher creatinine levels among Europeans (*p* < 0.01). Chest pain presentation differed significantly, with typical angina occurring more frequently in the European group (*p* = 0.02).

### 3.2. Pharmacological Therapy

Patients were treated according to current cardiovascular prevention practices. Pharmacological management consists of antihypertensive medications, which represent all principal guideline-recommended classes, and statins (Table 2). No stratified comparison of pharmacological therapy between race groups was performed because of incomplete quantitative data.

### 3.3. Coronary Artery Calcification and CT Findings by Race

**Figure 2:**
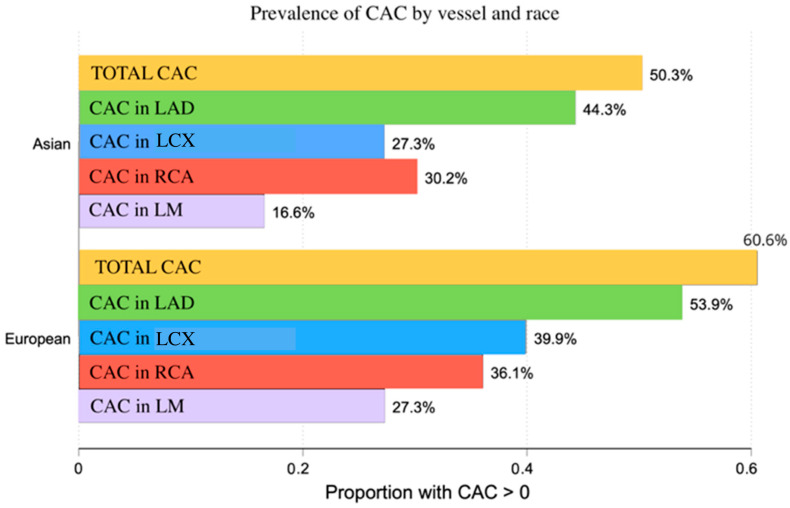
Grouped horizontal bar chart: prevalence of coronary artery calcification (CAC) by vessel and race. CAC was quantified in the left anterior descending (LAD), left circumflex (LCX), and right coronary artery (RCA).

Coronary artery calcification burden and coronary CT findings differed significantly between race groups (Table 3). The prevalence of CAC > 0 was 50.3% among Asian participants and 60.6% among European participants (*p* < 0.01), and total CAC scores were higher in the European group. Although median CAC values in individual coronary arteries were zero in both race groups, the prevalence of artery-specific CAC > 0 was consistently higher among European participants (Figure 2).

CAC in the LAD was present in 53.9% of Europeans and 44.3% of Asians (*p* < 0.01), CAC in the LCX in 39.9% and 27.3%, respectively (*p* < 0.01), CAC in the RCA in 36.1% and 30.2% (*p* = 0.03), and CAC in the LM in 27.3% and 16.6% (*p* < 0.01).

Significant coronary stenosis (≥50%) was observed in the LCX in 7.2% of Asian participants and 11.9% of European participants, in the RCA in 8.4% and 25.8%, respectively, and in the LM in 1.9% and 5.1%, respectively (all *p* < 0.01). Multivessel coronary stenosis (≥2 vessels) was more frequent among European participants (15.4%) than among Asian participants (9.5%) (*p* < 0.01). Calcified plaques were more frequently observed among European participants, whereas non-calcified plaques predominated among Asian participants across all coronary vessels.

### 3.4. Baseline Characteristics and Cardiovascular Risk Profile According to Continuous and Dichotomized Artery Calcium (CAC) Score

In bivariate analyses, continuous CAC score was significantly associated with age, sex, race, body mass index (BMI), SBP and DBP, hypertension, diabetes, statin use, plasma glucose, and creatinine levels (all *p* ≤ 0.02) (Table 4). In contrast, a dichotomous CAC score (CAC > 0) was associated with tobacco use (*p* = 0.02), alcohol consumption (*p* = 0.01), diabetes (*p* < 0.01), glucose (*p* < 0.01), and triglyceride concentration levels (*p* = 0.04). On the basis of bivariate analysis, a significant association was identified between CAC score and coronary CT findings, including the presence and extent of coronary stenosis, the number of vessels with significant stenosis, and plaque characteristics (Table 5).

### 3.5. Multivariable Linear Regression Analysis of Factors Associated with Coronary Artery Calcium Burden Among Patients with CAC > 0

**Figure 3:**
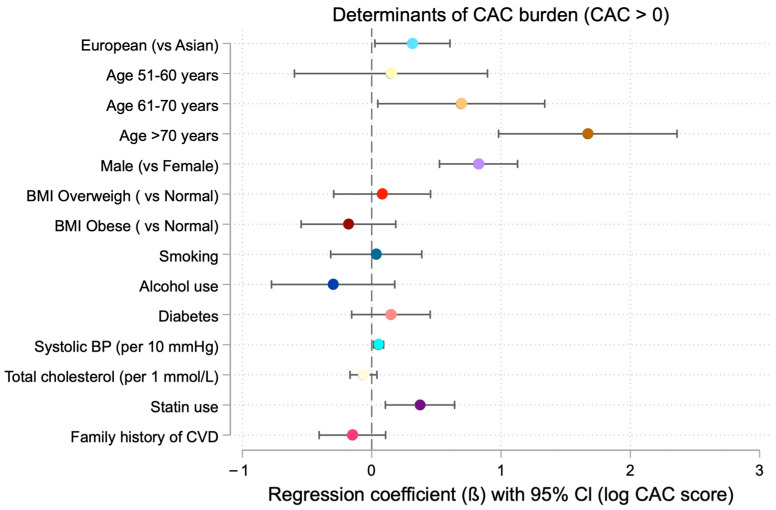
Determinants of CAC burden (CAC > 0) based on multivariable regression analysis. Covariates included race (light blue), age (yellow/orange/brown), sex (light purple), BMI (red and crimson), smoking (turquoise), alcohol use (dark blue), diabetes (pink), systolic blood pressure (SBP) (light turquoise), total cholesterol (pale yellow), statin use (dark purple), and family history of CVD (vvid crimson).

In the fully adjusted logistic regression model including BMI, smoking status, alcohol use, diabetes, systolic blood pressure category, total cholesterol category, statin use, and family history of CHD, European ethnicity remained independently associated with CAC presence (OR 1.38, 95% CI 1.04–1.83, *p* = 0.02) (Table 6). Among participants with CAC > 0, multivariate linear regression analysis demonstrated a significantly higher CAC burden in Europeans, with an average increase of 36.98% in CAC score compared with Asians (*p* = 0.02) (Table 7, Figure 3).

**Figure 4:**
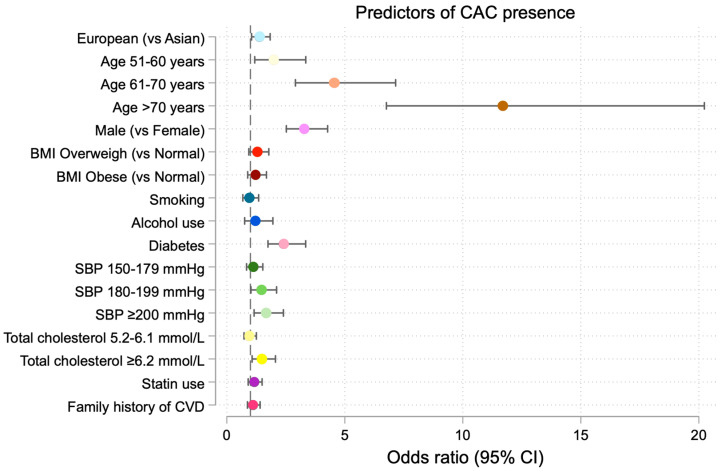
Predictors of CAC presence. Variables included race, age, sex, BMI, smoking, alcohol use, diabetes, systolic blood pressure (SBP), total cholesterol, statin use, and family history of CVD. Odds ratios (ORs) with 95% confidence intervals (Cis) are displayed for each predictor.

Male sex was strongly associated with CAC presence, with the odds of CAC > 0 being more than three times higher among males compared with females (OR = 3.24; 95% CI, 2.53–4.14; *p* < 0.001). Both the odds of CAC presence and CAC burden increased significantly across age categories (*p* < 0.001). Higher systolic blood pressure and higher total cholesterol levels were positively associated with both CAC presence (Figure 4) and CAC burden in multivariate models.

Statin use was positively associated with CAC burden, whereas family history of cardiovascular disease was not independently associated with CAC outcomes.

## 4. Discussion

This retrospective study, conducted at two tertiary medical centers in Kazakhstan, aimed to evaluate suspected coronary artery disease. Within this referral-based cohort, differences in plaque characteristics were observed between individuals classified as Asian and European participants.

Previous studies have reported variability in coronary calcium burden across different populations, including an investigation exploring genetic susceptibility to atherosclerotic plaque development [17]. In a complementary study, findings demonstrated that lipoprotein(a) (Lp(a)) levels were associated with coronary heart disease and stroke subtypes and varied by ancestry [18]. However, the present study was not designed to evaluate genetic features, and the observed associations may reflect multifactorial influences within a referral-based cohort. Therefore, these findings should not be interpreted as evidence of intrinsic biological differences.

The main finding of this study is that individuals classified as European ethnicity demonstrated a higher prevalence of coronary artery calcification compared with individuals classified as Asian ethnicity, who more frequently exhibited non-calcified and mixed plaques. In addition, calcified plaques were more commonly observed in the left main and right coronary arteries in the European group.

However, these observations represent associations within a clinical population undergoing CCTA and should therefore be interpreted within this context. Differences in cardiovascular risk factor distribution, referral patterns, and potential residual confounding may have influenced the observed findings.

Analysis of modifiable cardiovascular risk factors showed that individuals classified as European ethnicity had a higher prevalence of hypertension, obesity, smoking, alcohol consumption, and elevated creatinine levels, which likely contributed to the greater coronary calcification burden observed in this group.

In the present cohort, statin use was positively associated with CAC burden. These findings are consistent with prior literature suggesting potential associations between statin therapy and plaque characteristics [19]. However, the lack of detailed information regarding treatment duration and adherence limits the interpretation of this finding. Furthermore, recent studies have highlighted that high-resolution CCTA enables detailed visualization of coronary arteries and plaque morphology in low- to intermediate-risk patients, thereby supplying personalized risk stratification [20].

The differences in plaque phenotypes observed in this cohort may provide a basis for future research integrating artificial intelligence-based (AI-based) approaches and potential biochemical biomarkers, such as low-density lipoprotein receptor-1 (LOX-1), to further refine cardiovascular risk assessment [21]. However, the present cross-sectional study did not evaluate predictive modeling, biomarker levels, or longitudinal outcomes. These considerations remain exploratory and were not evaluated within the current study design.

The study has several limitations. The study population consisted of patients referred for coronary CT angiography at two tertiary centers, which may introduce referral bias and limit generalizability beyond the studied cohort. The extended study period (2008–2025) and the use of multiple CT scanners may have introduced measurement heterogeneity. In addition, residual confounding from unmeasured variables, including detailed treatment duration and cumulative medication exposure, cannot be excluded.

This study is limited by its retrospective and cross-sectional design, which restricts the ability to establish causal relationships and limits interpretation of associations with revascularization procedures and invasive coronary angiography findings. Furthermore, the absence of longitudinal outcome data precludes evaluation of the relationship between plaque characteristics and subsequent cardiovascular events. Access to comprehensive quantitative pharmacological data, particularly regarding antihypertensive drug classes, was also limited.

Risk stratification was based on selected clinical and CT angiographic parameters and relied on a modified ESC-based framework. To address these limitations, further longitudinal studies in more diverse populations are needed to validate the present findings. Prospective outcome-based investigations are required to determine the prognostic significance of the observed imaging differences.

## 5. Conclusions

This study identified differences in plaque characteristics between the studied groups within a clinical CCTA cohort. These findings represent descriptive associations derived from a retrospective cross-sectional design and should be interpreted in the context of multifactorial influences, including cardiometabolic risk factor distribution and environmental exposures. Prospective longitudinal studies with outcome data are needed to determine the prognostic implications of these imaging findings.

## Figures and Tables

**Table 1:** Overall descriptive statistics of participants by race.

Variables	Asian n = 1203	European n = 388	*p*-Value
Age (years)	58.9 ± 11.1	60.0 ± 10.9	0.05 *
Categorical Age (%)			
≤50	139 (11.6)	30 (7.8)	<0.01 **
51–60	197 (16.4)	38 (9.8)	
61–70	703 (58.4)	253 (65.2)	
>70	164 (13.6)	67 (17.3)	
Female (%)	470 (39.1)	156 (40.2)	0.69 **
Male (%)	733 (60.9)	232 (59.8)	
BMI Normal (%)	259 (21.5)	69 (17.8)	<0.01 **
BMI Overweight (%)	468 (38.9)	122 (31.5)	
BMI Obese (%)	476 (39.6)	196 (50.7)	
Systolic BP, mmHg	155.5 ± 34.8	160.8 ± 36.8	0.01 *
Diastolic BP, mmHg	91.3 ± 13.7	93.5 ± 14.1	<0.01 *
Diabetes (%)	202 (16.8)	76 (19.6)	0.21 **
Hypertension (%)	983 (81.8)	316 (81.4)	0.88 **
Smoking (%)	177 (14.7)	93 (23.9)	<0.01 **
Alcohol use (%)	74 (6.2)	43 (11.1)	<0.01 **
Family History of CVD (%)	552 (45.9)	168 (43.3)	0.37 **
Statin Use (%)	595 (49.5)	149 (38.4)	<0.01 **
Chest pain (%)			
Atypical	277 (23.0)	84 (21.7)	0.02 **
Typical	448 (37.2)	174 (44.9)	
Non-anginal	478 (39.7)	130 (33.5)	
Plasma glucose, mmol/L	5.3 ± 2.5	5.2 ± 2.1	0.47 *
Plasma fibrinogen level, g/L	3.2 ± 0.9	3.1 ± 0.9	0.44 *
Serum creatinine, µmol/L	71.5 (60–85)	80 (66–93)	<0.01 ***
Total Cholesterol, mmol/L	5.1 ± 1.2	4.9 ± 1.2	0.59 *
LDL-C, mmol/L	3.4 ± 1.1	3.3 ± 1.1	0.34 *
HDL-C, mmol/L	1.2 (1.0–1.5)	1.2 (0.9–1.5)	0.60 ***
Triglyceride concentration, mmol/L	1.4 (1.0–2.0)	1.4 (1.1–1.9)	0.23 ***

Percentages are represented in parentheses. Continuous variables are presented as mean ± SD or median (IQR), as appropriate; * Student’s *t*-test, ** Fisher’s exact test or Pearson’s chi-square test, *** Mann–Whitney U test.

**Table 2:** Pharmacological therapy was documented in the study cohort.

Pharmacological Class	Representative Agents
ACE inhibitors	Captopril, Perindopril, Ramipril
ARBs	Candesartan, Valsartan, Azilsartan
Dihydropyridines	Amlodipine, Felodipine
Diuretics	Hydrochlorothiazide, Indapamide
Potassium-sparing diuretics	Amiloride, Spironolactone
Beta-blockers	Atenolol, Bisoprolol, Metoprolol
Lipid-lowering therapy	Atorvastatin, Rosuvastatin

**Table 3:** Characteristics of coronary computed tomography (CT) findings by race.

Variables	Asian n = 1203	European n = 388	*p*-Value
CAC (total)			
Median (IQR)	0.2 (0–63.8)	14.7 (0–90.2)	<0.01 *
CAC > 0, n (%)	605 (50.3)	235 (60.6)	<0.01 **
CAC in LAD			
Median (IQR)	0 (0–32)	1.25 (0–38.5)	<0.01 *
CAC in LAD > 0, n (%)	533 (44.3)	209 (53.9)	<0.01 **
CAC in LCX			
Median (IQR)	0 (0–1.8)	0 (0–11.1)	<0.01 *
CAC in LCX > 0, n (%)	328 (27.3)	155 (39.9)	<0.01 **
CAC in RCA			
Median (IQR)	0 (0–1.6)	0 (0–4)	0.03 *
CAC in RCA > 0, n (%)	363 (30.2)	140 (36.1)	0.03 **
CAC in LM			
Median (IQR)	0 (0–0)	0 (0–0.9)	<0.01 *
CAC in LM > 0, n (%)	199 (16.6)	106 (27.3)	<0.01 **
Number of vessels with Stenosis ≥ 50%			
None	895 (74.4)	241 (62.1)	<0.01 **
Only One	194 (16.1)	87 (22.4)	
Only Two	71 (5.9)	35 (9.0)	
Three or LMD	43 (3.6)	25 (6.4)	
Stenosis of LAD			
None or <50%	938 (77.9)	302 (77.8)	0.95 **
≥50%	265 (22.0)	86 (22.2)	
Stenosis of LCX			
None or <50%	1116 (92.8)	342 (88.1)	<0.01 **
≥50%	87 (7.2)	46 (11.9)	
Stenosis of RCA			
None or <50%	1101 (91.6)	288 (74.2)	<0.01 **
≥50%	101 (8.4)	100 (25.8)	
Stenosis of LM			
None or <50%	1171 (97.3)	368 (94.9)	<0.01 **
≥50%	32 (1.9)	20 (5.1)	
LAD plaque present			
Non-calcified	636 (52.9)	183 (47.2)	0.01 **
Low-density, Non-calcified	83 (6.9)	18 (4.6)	
Calcified	484 (40.2)	187 (48.2)	
CX plaque present			
Non-calcified	871 (72.4)	211 (54.4)	<0.01 **
Low-density, Non-calcified	50 (4.2)	18 (4.6)	
Calcified	282 (23.4)	159 (40.9)	
RCA plaque present			
Non-calcified	828 (68.8)	229 (59.0)	<0.01 **
Low-density, Non-calcified	66 (5.5)	23 (5.9)	
Calcified	309 (25.7)	136 (35.1)	
LM plaque present			
Non-calcified	823 (68.4)	222 (57.2)	<0.01 **
Low-density, Non-calcified	50 (4.2)	13 (3.4)	
Calcified	330 (27.4)	153 (39.4)	

Percentages are represented in parentheses. Continuous variables are represented as median (IQR); * Mann–Whitney U test, ** Pearson’s chi-square test or Fisher’s exact test.

**Table 4:** Bivariate analysis for continuous and dichotomous coronary artery calcification (CAC) score (CAC score = 0 and CAC score > 0) with demographic variables and cardiovascular risk factors.

Variables	Continuous CAC Score	*p*-Value	CAC Score = 0 (n = 751)	CAC Score > 0 (n = 840)	*p*-Value
Age					
≤50	0 (0–0)	<0.01 *	134 (79.3)	35 (20.7)	<0.01 **
51–60	0 (0–12)		143 (60.9)	92 (39.2)	
61–70	6.7 (0–68.1)		416 (43.5)	540 (56.5)	
>70	63.9 (0–295)		58 (25.1)	173 (74.9)	
Female	0 (0–21)	<0.01 ***	372 (59.4)	254 (40.6)	<0.01 **
Male	13.9 (0–107.7)		379 (39.3)	586 (60.7)	
Asian	0.2 (0–63.8)	<0.01 ***	598 (49.7)	605 (50.3)	<0.01 **
European	14.7 (0–90.2)		153 (39.4)	235 (60.6)	
BMI					
Normal	0 (0–43)	<0.01 *	180 (54.9)	148 (45.1)	<0.01 **
Overweight	3.7 (0–89)		274 (46.5)	316 (53.6)	
Obese	4.7 (0–66)		296 (44.1)	376 (55.9)	
SBP	0.21	<0.01 ****	150.3 ± 34.4	162.5 ± 35.3	<0.01 ***
DBP	0.16	<0.01 ****	89.8 ± 13.5	93.6 ± 13.8	<0.01 ***
Hypertension					
No	0 (0–16)	0.01 ***	175 (60.1)	116 (39.9)	<0.01 **
Yes	5 (0–89.1)		576 (44.3)	723 (55.7)	
Smoking					
No	1 (0–68)	0.98 ***	641 (48.5)	680 (51.5)	0.02 **
Yes	12.1 (0–72.2)		110 (40.7)	160 (59.3)	
Alcohol use					
No	1.1 (0–75.6)	0.34 ***	709 (48.1)	765 (51.9)	0.01 **
Yes	17.2 (0–55.4)		42 (35.9)	75 (64.1)	
Family history of CHD					
No	4 (0–83)	0.02 ***	402 (46.2)	469 (53.8)	0.36 **
Yes	0.8 (0–59.7)		349 (48.5)	371 (51.5)	
Chest pain					
Atypical	10 (0–81.6)	<0.01 *	145 (40.2)	216 (59.8)	<0.01 **
Typical	3.3 (0–79.2)		287 (46.1)	335 (53.9)	
Non-anginal	0 (0–54.5)		319 (52.5)	289 (47.5)	
Use of Statins					
No	0.45 (0–48.4)	<0.01 ***	418 (49.4)	428 (50.6)	0.06 **
Yes	5.15 (0–97.9)		332 (44.6)	412 (55.4)	
Diabetes					
No	0 (0–55.4)	0.01 ***	668 (50.9)	645 (49.1)	<0.01 **
Yes	22.9 (0–151.6)		83 (29.9)	195 (70.1)	
Plasma glucose	0.09	0.02 ****	5.0 ± 2.3	5.6 ± 2.4	<0.01 ***
Plasma fibrinogen	0.02	0.50 ****	3.1 ± 0.9	3.2 ± 1.0	0.23 ***
Serum creatinine	0.15	0.01 ****	70 (58–84)	77 (64–89)	<0.01 ***
Total Cholesterol	−0.02	0.42 ****	5.1 ± 1.2	5.1 ± 1.3	0.83 ***
LDL-C	−0.05	0.09 ****	3.4 ± 1.1	3.3 ± 1.1	0.44 ***
HDL-C	−0.09	<0.01 ****	1.2 (1.1–1.5)	1.2 (1.0–1.5)	0.01 ***
Triglyceride concentration	0.07	0.02 ****	1.3 (1.0–1.8)	1.4 (1.0–2.0)	0.04 ***

Percentages are represented in parentheses. Continuous variables are presented as mean ± SD or median (IQR), as appropriate; * Kruskal–Wallis test, ** Pearson’s chi-square test or Fisher’s exact test, *** Two-sample *t*-test or Mann–Whitney U test, **** Spearman’s correlation coefficient.

**Table 5:** Bivariate analysis for continuous and dichotomous CAC score (CAC score = 0 and CAC score > 0) with coronary CT variables.

Variables	Continuous CAC Score	*p*-Value	CAC Score = 0 (n = 751)	CAC Score > 0 (n = 840)	*p*-Value
Stenosis of LAD					
None or <50%	0 (0–19)	<0.01 ***	717 (57.8)	523 (42.2)	<0.01 **
≥50%	153 (46.5–523.2)		34 (9.7)	317 (90.3)	
Stenosis of LCX					
None or <50%	0 (0–45.7)	<0.01 ***	738 (50.6)	720 (49.4)	<0.01 **
≥50%	319 (67–834.2)		13 (9.8)	120 (90.2)	
Stenosis of RCA					
None or <50%	0 (0–45)	<0.01 ***	729 (52.5)	660 (47.5)	<0.01 **
≥50%	151.6 (21.8–701.4)		21 (10.5)	180 (89.6)	
Stenosis of LM					
None or <50%	1 (0–66.1)	<0.01 ***	749 (48.7)	790 (51.3)	<0.01 **
≥50%	422.8 (206–1016)		2 (10.5)	17 (89.5)	
Number of vessels with Stenosis ≥ 50%					
None	0 (0–14)	<0.01 *	695 (61.2)	441 (38.8)	<0.01 **
Only One	55.2 (16.2–160.7)		46 (16.4)	235 (83.6)	
Only Two	313.5 (69.4–668.8)		6 (5.7)	100 (94.3)	
Three or LMD	697.7 (240–1120.5)		4 (5.9)	64 (94.1)	
LAD plaque present					
Non-calcified	0 (0–0)	<0.01 *	647 (79.0)	172 (21.0)	<0.01 **
Low-density, Non-calcified	0 (0–7.7)		62 (61.4)	39 (38.6)	
Calcified	76 (20–269)		42 (6.3)	629 (93.7)	
CX plaque present					
Non-calcified	0 (0–11.4)	<0.01 *	692 (63.9)	390 (36.1)	<0.01 **
Low-density, Non-calcified	0 (0–28.5)		38 (55.9)	30 (44.1)	
Calcified	102.5 (28.4–386)		21 (4.8)	420 (95.2)	
RCA plaque present					
Non-calcified	0 (0–14.7)	<0.01 *	672 (63.6)	385 (36.4)	<0.01 **
Low-density, Non-calcified	0 (0–19.8)		48 (53.9)	41 (46.1)	
Calcified	114.6 (24.9–382)		31 (6.9)	414 (93.1)	
LM plaque present					
Non-calcified	0 (0–10)	<0.01 *	693 (66.3)	352 (33.7)	<0.01 **
Low-density, Non-calcified	0 (0–9.7)		39 (61.9)	24 (38.1)	
Calcified	82 (20.4–313)		19 (3.9)	464 (96.1)	

Percentages are represented in parentheses. Continuous variables are represented as median (IQR); * Kruskal–Wallis test, ** Pearson’s chi-square test or Fisher’s exact test, *** Mann–Whitney U test.

**Table 6:** Odds ratio of CAC presence (CAC score > 0) by independent variables in multivariate logistic regression.

Variable	OR (95% CI)	*p*-Value
Asian	1.00 (Reference)	0.02
European	1.39 (1.05–1.83)	
Age		
≤50	1.00 (Reference)	<0.01
51–60	1.98 (1.18–3.34)	
61–70	4.55 (2.91–7.15)	
>70	11.69 (6.76–20.23)	
Female	1.00 (Reference)	<0.01
Male	3.28 (2.52–4.27)	
BMI		
Normal	1.00 (Reference)	0.13
Overweight	1.28 (0.93–1.77)	
Obese	1.22 (0.88–1.68)	
Smoking		
No	1.00 (Reference)	0.79
Yes	0.96 (0.68–1.35)	
Alcohol use		
No	1.00 (Reference)	0.80
Yes	1.21 (0.75–1.95)	
Diabetes		
No	1.00 (Reference)	<0.01
Yes	2.41 (1.74–3.34)	
Family history of CVD		
No	1.00 (Reference)	0.39
Yes	1.11 (0.87–1.41)	
Use of Statins		
No	1.00 (Reference)	0.23
Yes	1.16 (0.91–1.49)	
SBP *		
<150 mm Hg	1.00 (Reference)	<0.01
150–179 mm Hg	1.13 (0.84–1.53)	
180–199 mm Hg	1.46 (1.02–2.11)	
≥200 mm Hg	1.66 (1.15–2.39)	
Total cholesterol *		
Below 5.2 mmol/L	1.00 (Reference)	0.02
5.2–6.1 mmol/L	0.95 (0.72–1.25)	
6.2 and above	1.48 (1.07–2.06)	
Intercept	0.06 (0.04–0.11)	<0.01

* Due to violation of the linearity assumption, SBP and total cholesterol were categorized to meet the assumption.

**Table 7:** Multivariate linear regression coefficients for independent variables of the CAC score among people with a CAC score > 0.

Variable	Coefficient (95% CI) (Log of CAC Score)	Corresponding Percentage Change in CAC Score (95% CI)	*p*-Value
Asian	Reference	Reference	0.03
European	0.31 (0.02–0.60)	36.98% (2.51% to 83.09%)	
Age			
≤50	Reference	Reference	<0.01
51–60	0.15 (−0.59–0.89)	16.06% (−44.97% to 144.83%)	
61–70	0.69 (0.05–1.34)	99.81% (4.80% to 280.97%)	
>70	1.67 (0.98–2.36)	432.37% (166.71% to 959.69%)	
Female	Reference	Reference	<0.01
Male	0.82 (0.52–1.13)	128.44% (68.95% to 209.01%)	
BMI			
Normal	Reference	Reference	0.02
Overweight	0.08 (−0.29–0.45)	8.40% (−25.45% to 57.65%)	
Obese	−0.18 (−0.54–0.18)	−16.47% (−42.03% to 20.39%)	
Smoking			
No	Reference	Reference	0.85
Yes	0.03 (−0.32–0.38)	3.55% (−27.16% to 47.26%)	
Alcohol use			
No	Reference	Reference	0.23
Yes	−0.29 (−0.77–0.17)	−25.79% (−53.92% to 19.50%)	
Diabetes			
No	Reference	Reference	0.34
Yes	0.15 (−0.15–0.45)	16.03% (−14.35% to 57.20%)	
Family history of CVD			
No	Reference	Reference	0.25
Yes	−0.15 (−0.41–0.11)	−13.89% (−33.42% to 11.36%)	
Use of Statins			
No	Reference	Reference	<0.01
Yes	0.37 (0.11–0.64)	45.25% (11.15% to 89.83%)	
SBP, per 10 mm Hg	0.005 (0.001–0.009)	0.53% (0.14% to 0.92%)	<0.01
Total cholesterol, per 1 mmol/L	−0.06 (−0.16–0.04)	−6.15% (−15.39% to 4.13%)	0.23
Intercept	1.89 (0.84–2.94)		<0.01

## Data Availability

The original contributions presented in this study are included in the article. Further inquiries can be directed to the corresponding author.

## Abbreviations

The following abbreviations are used in this manuscript:

ACEi	Angiotensin-Converting Enzyme inhibitors
AI	Artificial Intelligence
ARBs	Angiotensin II Receptor Blockers
ASCI-PT	Asian Society of Cardiovascular Imaging-Practical Tutorial
BMI	Body Mass Index
CAC	Coronary Artery Calcium
CAD	Coronary Artery Disease
CCBs	Calcium Channel Blockers
CCTA	Coronary Computed Tomography Angiography
CHD	Coronary Heart Disease
CI	Confidence Intervals
CT	Computed Tomography
CVD	Cardiovascular Disease
DBP	Diastolic Blood Pressure
ESC	European Society of Cardiology
EU	European Union
GBD	Global Burden of Disease
HDL-C	High-density Lipoprotein Cholesterol
HU	Hounsfield Units
IHD	Ischemic Heart Disease
LOX-1	Lectin-like Oxidized Low-Density Lipoprotein Receptor-1
Lp (a)	Lipoprotein (a)
IQR	Interquartile Range
LAD	Left Anterior Descending Artery
LCX	Left Circumflex Artery
LDL-C	Low-Density Lipoprotein Cholesterol
LM	Left Main Coronary Artery
MESA	Multi-Ethnic Study of Atherosclerosis
MACE	Major Adverse Cardiovascular Events
OR	Odds Ratio
RCA	Right Coronary Artery
SBP	Systolic Blood Pressure
SD	Standard Deviations
